# 1147. Sentinel Surveillance of Bacterial Pneumonia in Children Under 5 years Treated in HOMI - Fundación Hospital pediatrico la Misericordia in Bogotá, Colombia 2016-2020

**DOI:** 10.1093/ofid/ofab466.1340

**Published:** 2021-12-04

**Authors:** German Camacho Moreno, Carolina Duarte Valderrama, Jacqueline Palacios, Luz Angela Calvo, Ivy Talavera, Jaime Moreno Castañeda, Luz Yanet Maldonado Cortes, Daniela Jerez, Carolina Garcia Romero, Karen Jimenez Rodriguez, Olga Sanabria, Yenny Marcela Elizalde Rodriguez, Leidy Monroy, Maria Cristina Duarte

**Affiliations:** 1 Universidad Nacional de Colombia - Fundacion HOMI - Hospital Infantil Universitario San José, Bogotá, Distrito Capital de Bogota, Colombia; 2 Instituto Nacional de Salud, Bogotá, Distrito Capital de Bogota, Colombia; 3 Ministerio de Salud, Bogota, Distrito Capital de Bogota, Colombia; 4 Secretaria de Salud de Bogota, Bogota, Distrito Capital de Bogota, Colombia; 5 Organización Panamericana de la Salud, Bogota, Distrito Capital de Bogota, Colombia; 6 HOMI, Fundacion Hospital pediatrico de la Misericordia, Bogota, Distrito Capital de Bogota, Colombia; 7 HOMI, Fundación Hospital pediatrico de la Misericordia, Bogota, Distrito Capital de Bogota, Colombia

## Abstract

**Background:**

Pneumonia is one of the leading causes of hospitalization and death in children under 5y. The main causes of bacterial pneumonia (BP) are *Streptococcus pneumoniae* (Spn) and *Haemophilus influenzae* (Hi). Colombia implemented the Hib vaccine in 1997 with a 3 + 0 scheme and the PCV10 vaccine in 2012, using a 2 + 1 scheme. Sentinel surveillance of BP is carried out at HOMI - Fundación Hospital Pediátrico La Misericordia, which is part of the invasive bacterial vaccine preventable disease surveillance network.

**Methods:**

A daily active search for cases that met the definitions established in the protocol of the Pan American Health Organization was carried out. All hospitalized patients under 5 years of age with a diagnosis of community acquired pneumonia (ICD10 J10 to J22) were classified as suspected cases, while all suspected cases in which chest X-ray showed a radiological pattern compatible with bacterial pneumonia were considered a probable case. Blood cultures were taken from probable cases; if results were positive (Spn, Hi), the samples were sent to the district and national reference laboratories for confirmation and serotyping. The data obtained in the period January 2016 to December 2020 were analyzed.

**Results:**

5272 suspected cases of bacterial pneumonia were found, of which 60% were < 2 y. The highest incidence occurred from March to June (Figure 1). Blood cultures were performed in 2223 (92%) of the 2432 (46.1%) probable cases, confirming 127 (5.2%) cases. Spn, Hi, and other bacteria were found in 55, 27, and 28 cases, respectively (Table 1). Serotyping was performed in 85.4% of the Spn isolates and 77.7% of Hi isolates. The most frequent Spn serotypes were Spn19A in 19 cases (40.4%), Spn3 in 12 cases (25.5%), and Spn14 in 4 cases (8.5%). The presence of Spn19A has increased over time (Figure 2). The most frequent Hi was non-typeable in 13 patients (61.9%), followed by serotype b 6 (28.5%) and serotype a 2 (9.5%). The rate of hospitalization for BP was 9/1000 children < 5 years, and 43 patients died. Case fatality rate was 1.7% among probable cases.

Graph 1. Trend of suspected bacterial pneumonia cases in children under 5 years old. HOMI. 2016-2020

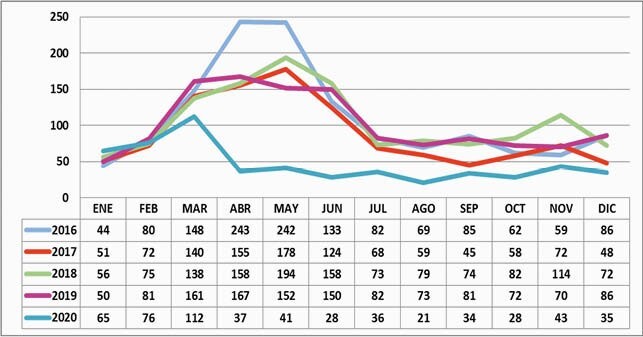

Table 1. Bacterial pneumonia isolates. HOMI. 2016 - 2020

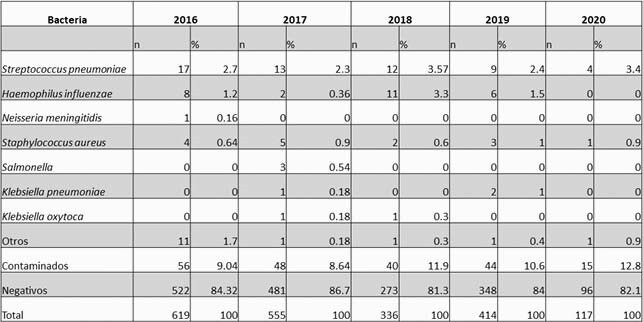

Graph 2. Bacterial pneumonia serotypes. HOMI. January 2016 - December 2020

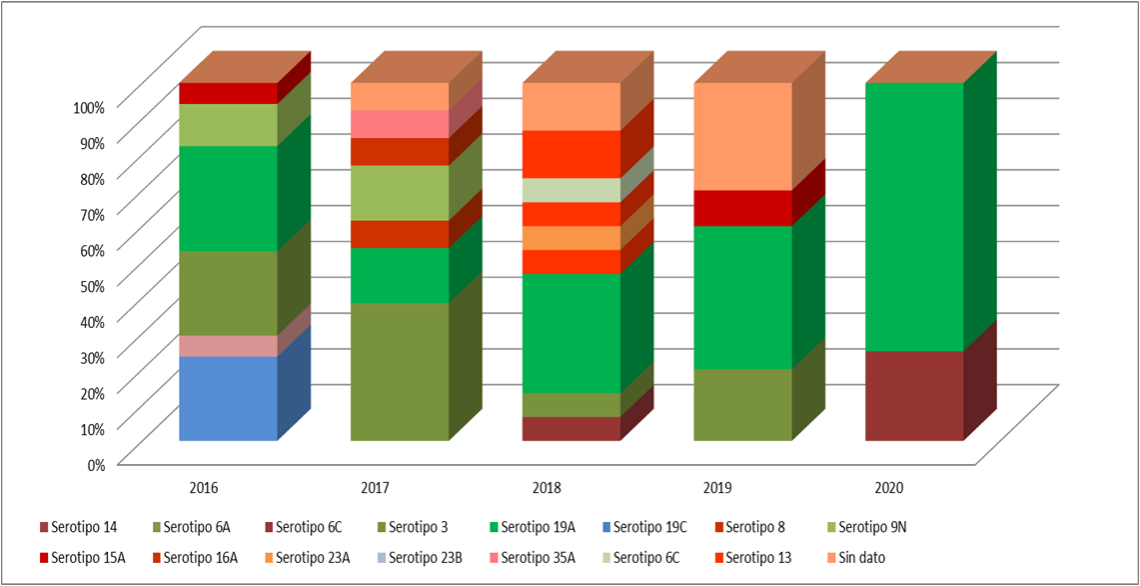

**Conclusion:**

BP mainly occurs in 2-year-old children. Spn 19A is the most common bacteria. Although the most frequent Hi is non-typeable, cases of Hib are still observed. Sentinel surveillance allows measuring the impact of public health interventions on this disease.

**Disclosures:**

**German Camacho Moreno, n/a**, **Pfizer and MSD** (Research Grant or Support, Speaker’s Bureau, Other Financial or Material Support, Has received support from Pfizer for participation in congresses)

